# High-Resolution X-Ray Techniques as New Tool to Investigate the 3D Vascularization of Engineered-Bone Tissue

**DOI:** 10.3389/fbioe.2015.00133

**Published:** 2015-09-07

**Authors:** Inna Bukreeva, Michela Fratini, Gaetano Campi, Daniele Pelliccia, Raffaele Spanò, Giuliana Tromba, Francesco Brun, Manfred Burghammer, Marco Grilli, Ranieri Cancedda, Alessia Cedola, Maddalena Mastrogiacomo

**Affiliations:** ^1^Consiglio Nazionale delle Ricerche – Istituto NANOTEC, c/o Dipartimento di Fisica, Università Sapienza, Rome, Italy; ^2^Department of Science, Roma Tre University, Rome, Italy; ^3^Istituto di Cristallografia, Consiglio Nazionale delle Ricerche, Rome, Italy; ^4^School of Applied Sciences, RMIT University, Melbourne, VIC, Australia; ^5^Australian Synchrotron, Clayton, VIC, Australia; ^6^School of Physics and Astronomy, Monash University, Clayton, VIC, Australia; ^7^Dipartimento di Medicina Sperimentale dell’Università di Genova, AOU San Martino-IST, Genova, Italy; ^8^Elettra – Synchrotron Radiation Trieste S.C.p.A, Trieste, Italy; ^9^Dipartimento di Ingegneria e Architettura, Università di Trieste, Trieste, Italy; ^10^European Synchrotron Radiation Facility, Grenoble, France; ^11^Department of Analytical Chemistry, Ghent University, Ghent, Belgium; ^12^Dipartimento di Fisica, Università Sapienza, Rome, Italy; ^13^Consiglio Nazionale delle Ricerche – Istituto dei Sistemi Complessi, c/o Dipartimento di Fisica, Università Sapienza, Rome, Italy

**Keywords:** X-ray phase-contrast tomography, tissue engineering, vascularization, X-ray micro-diffraction, bone tissue

## Abstract

The understanding of structure–function relationships in normal and pathologic mammalian tissues is at the basis of a tissue engineering (TE) approach for the development of biological substitutes to restore or improve tissue function. In this framework, it is interesting to investigate engineered bone tissue, formed when porous ceramic constructs are loaded with bone marrow stromal cells (BMSC) and implanted *in vivo*. To monitor the relation between bone formation and vascularization, it is important to achieve a detailed imaging and a quantitative description of the complete three-dimensional vascular network in such constructs. Here, we used synchrotron X-ray phase-contrast micro-tomography to visualize and analyze the three-dimensional micro-vascular networks in bone-engineered constructs, in an ectopic bone formation mouse-model. We compared samples seeded and not seeded with BMSC, as well as samples differently stained or unstained. Thanks to the high quality of the images, we investigated the 3D distribution of both vessels and collagen matrix and we obtained quantitative information for all different samples. We propose our approach as a tool for quantitative studies of angiogenesis in TE and for any pre-clinical investigation where a quantitative analysis of the vascular network is required.

## Introduction

Tissue Engineering (TE) is the biotechnology that combines aspects of medicine, biology, and engineering to generate, repair, or replace human tissues. In particular, the TE approach may be used to regenerate bone by implanting a porous ceramic scaffold combined with bone marrow stromal cells (BMSC) *in vivo*. The scaffold plays a fundamental role since it acts as a guide and it stimulates the growth thus creating TE constructs or living bio-composites (Hench and Polak, 2002; Cancedda et al., 2003). An ideal scaffold should present high porosity, maximum surface area for bone growth, and an interconnected pore space with pores having a sufficiently large size to allow the penetration and diffusion of the blood vessels (Quarto et al., 2001). Indeed, the efficiency of an artificially implanted construct depends on the timely delivery and exchange of nutrients (oxygen, glucose, amino acids, etc.) from surrounding blood vessels to the BMSC, and the contemporary removal of the metabolism waste products (CO_2_, lactate, and urea) (Carano and Filvaroff, 2003; Jain, 2003). Therefore, the control of the angiogenesis of the micro-vascular network with proper spatial organization is a key step to obtain tissue regeneration and repair (Carano and Filvaroff, 2003). Furthermore, a deeper understanding of the developmental neo-vascularization is necessary for a better treatment of many pathological conditions, including cancer, diabetes, psoriasis, and articular degeneration.

In this framework, the need to detect subtle changes in tissue microvasculature requires the use of minimally invasive and bulk-sensitive experimental techniques. Currently, conventional characterization techniques have limitations: 2D imaging, such as histology, yields incomplete spatial coverage with possible data misinterpretation, whereas conventional micro-computed tomography (micro-CT) does not achieve sufficient resolution and contrast. In particular, it is virtually impossible to observe blood vessels by conventional X-ray imaging techniques without using contrast agents (Plouraboue et al., 2004; Risser et al., 2007). Recently, new contrast agent method for the synchrotrons radiation X-ray imaging of organisms was developed. As example, the micro-bubble was applied as a phase-contrast agent for angiography applications (Tang et al., 2011). Thus, a 3D imaging spanning from a few millimeters to hundreds of nanometers, able to discriminate the smallest micro-capillaries and the volume of the scaffold with and without invasive contrast agent and without aggressive sample preparation, is extremely desirable.

Conventional X-ray radiography and tomography are well established tool for imaging the internal structure of thick objects, based on absorption properties of the sample. The average thickness and 3D distribution of newly formed bone, at different implantation times, as well as scaffold modifications occurring after implantation, have been largely investigated by synchrotron radiation techniques (Komlev et al., 2006; Mastrogiacomo et al., 2007). Other authors reported *in vivo* quantification of blood vessels in mice, ranging from largest to smallest structures using contrast-enhanced micro-CT for studying the physiological and pathological processes (Nebuloni et al., 2014).

Nevertheless, for weakly absorbing materials (like biological materials), the X-ray attenuation due to the sample becomes often too small to give detectable contrasts. A better contrast can be obtained by imaging the phase modulation induced by the sample in a coherent or partial coherent beam (Cloetens et al., 1996; Di Fonzo et al., 1998). In this case, the phase contrast can be up to 1000 times higher than the absorption contrast (Bravin et al., 2013). The tomography provides the additional benefit to discriminate the different depths inside the sample and to provide a map of the different layers.

In addition, the combination of different investigation techniques (Cedola et al., 2014) on the same sample provides a deeper understanding. In particular, the combination of X-ray micro-diffraction (XRμD) (Cedola et al., 2006) and synchrotron X-ray phase-contrast micro-tomography (Bravin et al., 2013) (XRPCμT) allows to combine the structural information of the regenerated bone system, obtained by XRμD, with the micrometer morphological information obtained by XRPCμT.

The X-ray diffraction is the main technique suitable for studying the atomic order in the matter. It is based on the constructive interference of hard X-ray scattered by the atomic distribution of the sample (Giacovazzo et al., 1993).

In this work, we use high-resolution in-line propagation XRPCμT for imaging the 3D vascular network in bone-engineered constructs, in an ectopic bone formation mouse-model using different staining preparation: (i) with MICROFIL^®^ (Flowtech, Inc., Carver, Massachusetts) (Zagorchev et al., 2010; Ehling et al., 2014); (ii) with phosphotungstic acid (PTA) (Descamps et al., 2014); (iii) without staining. The synchrotron XRPCμT was able to visualize the 3D vascularization network inside the scaffold, without any sample sectioning.

With the aim to provide additional structural information (Campi et al., 2012, 2013), and in order to characterize the well-packed collagen fibers inside the scaffold pores, the X-ray tomographic study was complemented with scanning XRμD. To show the applicability of the technique in different experimental conditions, this combined technique was applied to samples that underwent different staining procedures.

The high quality of the images enabled the extraction of a quantitative information for all the different sample preparations.

In this study, our goal was mainly methodological and we investigated by the combined technique only one sample for each experimental group. However, in addition to show the validity of the method, we were able to obtain also some biological information. Although a statistically significant biological study was far from the scope of the work, we were able to successfully demonstrate the effectiveness of the XRPCμT approach to understand angiogenesis in the engineered bone tissues representative of all experimental groups.

## Materials and Methods

### Sample preparation

All experimental animal procedures were carried out in the IRCCS AOU San Martino – IST Animal Facility (Genoa, Italy), in the respect of the national current regulations regarding the protection of animals used for scientific purpose (D.lgsvo 27/01/1992, n. 116). Research protocols have been evaluated and approved by the IRCCS AOU San Martino – IST *Ethical Committee for animal experimentation (CSEA)* as Animal use project n. 336 communicated to The Italian Ministry of Health, having regard to the article 7 of the D.lgs 116/92.

The studied scaffold was Skelite™ (Millenium Biologix Corp., Kingston, Canada), which is a bone graft substitute containing silicon in the form of Si-TCP, and consisting of approximately 67% Si-TCP and 33% HA/B-TCP. The scaffolds were seeded with *ex vivo* expanded sheep BMSC and implanted subcutaneously on the back of immuno-compromised mice (CD-1 nu/nu mice, females, Charles River) according to the methods previously described by Martin^14^.

### Cell isolation

Bone marrow aspirates were harvested from the posterior iliac crest of each animal (female sheep of Italian Biellese strain) under approval of the competent ethical committee and legal authorities. Bone marrow samples were washed twice with PBS (phosphate buffered saline). Marrow specimens were stained with a nuclear stain (0.1% methyl violet in 0.1 M citric acid) and the nucleated fraction was counted. Cells were suspended in Coon’s modified Ham’s F12 medium supplemented with 10% FCS, 100 IU/ml penicillin, and 100 mg/ml streptomycin and plated at a density of 1 × 10^6^ cells/cm^2^. Human recombinant fibroblast growth factor 2 (FGF2) (1 ng/ml) was added since the beginning of the culture. Medium was changed 2 days after the original plating and then twice a week. When cells in the culture dishes were nearly confluent (passage 0), BMSC were detached with 0.05% trypsin–0.01% EDTA and 5 × 10^5^ cells were replated in 100-mm dishes (passage 1). At the next confluence, BMSC were detached and used for implants in immuno-deficient (ID) (CD-1 nu/nu) mice.

### Cell seeding

After 3–4 weeks of *in vitro* expansion, sheep BMSC were detached from the Petri dishes with 0.05% trypsin and 0.01% EDTA, washed in serum-free medium, and resuspended at 2.5 × 10^6^ cells/20 μl of fibrinogen solution. The cell suspension was seeded onto each cube of biomaterial (3 mm × 3 mm × 3 mm) and 20 μl of thrombin were added (Tissucol, Baxter, Vienna, Austria). The polymerization reaction was allowed to proceed at 37°C for 10 min, then bioceramic/BMSC composites were subcutaneously implanted in ID mice.

### Animal surgery

Mice were cared and treated according to institutional guidelines approved by the IRCCS AOU San Martino – IST Ethical Committee for animal experimentation (CSEA), in the respect of the national current regulations regarding the protection of animals used for scientific purpose (D.lgs 27/01/1992, n. 116) and communicated to the Italian Ministry of Health, having regard to the article 7 of the D.lgs 116/92. Animals were anesthetized by intraperitoneal injection of ketamine (80–100 mg/kg) and xilazine (5–10 mg/kg). At the end of *the selected implantation time* scaffolds were removed from the host animals for analysis.

**Figure 1:**
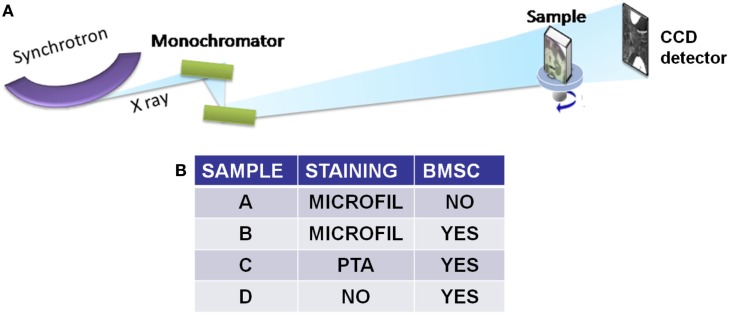
**(A)** Pictorial view of the experimental configurations used for XRPCμT measurements. The in-line, or propagation based, phase-contrast experimental setup, installed at the SLS synchrotron at PSI in Switzerland. **(B)** The table summarizes the characteristics of the different investigated samples.

All *in vivo* experiments were performed in triplicate, but only one sample for each group was XRPCμT analyzed. As control, one group of scaffolds was implanted without sheep BMSC pre-seeding. Four groups of mice were analyzed as reported in Table I in Figure 1B: (A) no BMSC pre-seeding and perfusion with MICROFIL^®^; (B) BMSC pre-seeding and perfusion with MICROFIL^®^, a low-viscosity radio opaque polymer (Flowtech, Inc., Carver, Massachusetts) well suited for vascularization studies; (C) BMSC pre-seeding and staining with PTA (PTA solution, Sigma-Aldrich Corp., St. Louis, MO, USA); (D) BMSC pre-seeding and perfusion with saline.

The animals perfused by MICROFIL^®^ at 4 weeks received an intraperitoneal injection of anesthetics (ketamine 80–100 mg/kg and xilazine 5–10 mg/kg) before the treatment. First, to eliminate the blood from the vessels, a solution of heparin 50 U/ml in 0.9% sodium chloride solution was perfused from the left ventricle until the outgoing liquid from the right atrium became clear. Then, a solution of MICROFIL^®^ was injected in the same way with a combination of MV compounds: MV diluent was mixed to MV-130 Red (4:5), with the final addiction of 1/20 of MV curing agent. At the end of perfusion, animals were sacrificed and transferred overnight at 4°C to allow the solidification of the MICROFIL^®^ agent. One day after the perfusion, the scaffolds were recovered from the animal, fixed with a solution of 4% paraformaldehyde for 2 days and maintained in 70% alcohol. For the saline perfusion, the same procedure was followed but the mice were perfused with 0.9% NaCl solution instead of the MICROFIL^®^ agent. For the PTA staining, the scaffolds recovered from the mice were directly placed in a solution containing 0.7% PTA diluted in 70% ethanol for 12 h and kept under slow rotation (Martin et al., 1997). The samples were then transferred to fresh 70% ethanol. For each experimental condition, after the XRPCμT analysis, a chosen sample was dehydrated in ethanol at increasing concentration, embedded in methylmethacrylate and transversally cut using a diamond saw (Gillings–Hamco, Hamco Machines, Inc., Rochester, NY, USA) in serial sections (≈150 μm thick) for the SXRμD measurements.

### X-ray phase-contrast tomography measurements

Among the different approaches for XRPCμT (Diemoz et al., 2012), in this work we used in-line free space propagation to reach high spatial resolution for the detection of micro-vessels. This technique is based on the interference fringes produced by the differently refracted beams crossing the sample. Therefore, this technique transforms the phase variation due to the object refraction index in intensity modulation directly detectable by a CCD camera. Suitable algorithms of phase retrieval allow to recover the object refraction index starting from the interference fringes produced by the object itself.

The experiment was carried out at TOMCAT beamline at the Swiss Light Source (SLS) in Villigen (Switzerland). The monochromatic incident X-ray energy was 24 keV and a CCD camera with a pixel size of about 0.64 microns was set at a distance of 5 cm from the sample. The tomography has been acquired with 1601 projections covering a total angle range of 360°. The setup does not comprise optical X-ray elements. For this reason, the spatial resolution is only limited by the detector resolution.

On the other hand, the image captured by in-line propagation always contains mixed absorption and phase-contrast effects. Therefore, specific algorithm must be used to decouple (at least in part) absorption from phase information (Paganin et al., 2002).

The phase retrieval algorithm proposed by Paganin et al. (2002) was applied to all projections of the tomographic measurements prior to the slices reconstruction.

### Quantitative analysis of X-ray phase-contrast tomography

Once the sample volume was reconstructed from the acquired projections, a stack of reconstructed slices of the sample was obtained. The quantitative analysis of the tomographic volumes, to obtain the number and section of the vessels as a function of the depth inside the sample (Plot 1 and Plot 2), was performed exploiting the software *ImageJ*. This software, developed for the image treatment, allows for the visualization and analysis of each reconstructed slice of the stack. The number of branches was calculated exploiting *Skeleton*, a plugin of *ImageJ*. Before applying *Skeleton*, a proper segmentation of the vessels (performed with the software *Volview*) was necessary.

Since within each group, preliminary analyses by histology had shown a high consistency in the data for all scaffolds, the XRPCμT analysis was performed on one selected scaffold for each of the four mice groups. In all cases, data obtained from the XRPCμT analysis of the selected scaffold were comparable to the data derived from the histology analysis of the other scaffolds of the group.

### Scanning X-ray micro-diffraction measurements

We used scanning XRμD for the investigation of the periodical assembly of collagen fibrils in the implanted constructs. Scaffold sections with a thickness of about 100 μm were measured on the ID13 beamline of the European Synchrotron Radiation Facility, ESRF, France. The scanning micro-diffraction setup was constituted by a double-crystal monochromator and a Kirkpatrick–Baez mirror as focusing system, producing a beam size of 1μm × 1 μm with a wavelength of 0.976 Å. 2D diffraction patterns were recorded in transmission by a FreLon CCD detector (2048 × 2048 pixels with size 50 μm^2^) placed at a distance of 110 cm from the sample with an acquisition time of 5s. In this way, we achieved a q range of [0.5–30] nm^−1^ to measure simultaneously the small-angle X-ray scattering and the wide angle X-ray scattering. 2D diffraction patterns have been radially and azimuthally integrated to provide 1D profiles of intensity, I(q), vs. transfer moment, q = 4πsin(θ)/λ, and angular intensity distribution I(Φ). We focused on the collagen signal collected at medium angle scattering around q = 5.6 nm^−1^. The samples were scanned by piezo-scanning stage with 0.1 micron repeatability.

The collagen equatorial reflection was modeled with a Gaussian added to second-order polynomial background. The peak position q_C_ gives the lateral spacing, D = 2π/q_C_, while the area under the peak accounts the total collagen mass. The orientation degree of the collagen molecules are given by the area under the peaks of the azimuthal profiles I(Φ). The total area under the I(Φ) curve is the sum of the area under the peaks, A_Φ_, which is proportional to the fraction of aligned molecules, and the area under the constant background (dotted line), A_BKG_, which is proportional to the fraction of randomly oriented molecules. The orientation degree, corresponding to the fraction of the aligned molecules can be defined as the ratio A_Φ_/A_BKG_.

## Results

### XRPCμCT analysis

**Figure 2:**
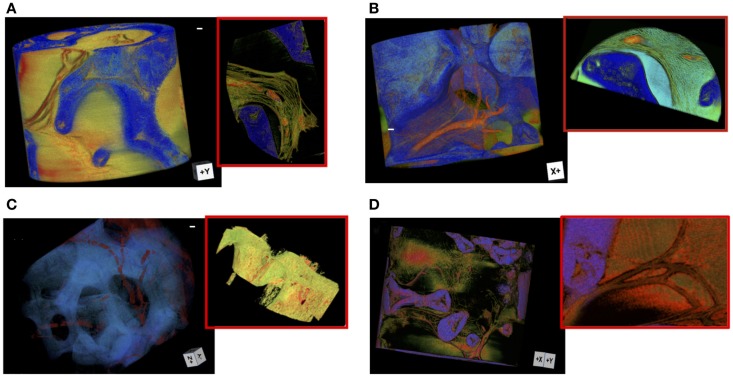
**Vessels distributions inside four different samples implanted for 4 weeks in the mice**. The size-bar corresponds to 30 μm. The first two samples, A and B, were both prepared with MICROFILL^®^ but, while the sample A was not pre-seeded with BMSCs before implantation, the sample B was pre-seeded with BMSCs. The sample C was also pre-seeded with BMSCs, but after the recovery of the scaffold from the animal it was stained with PTA. The sample D was a BMSC seeded not stained sample. **(A)** The vessels in the sample A are rendered in red, the scaffold in blue, and the soft tissue in yellow and green. The inset shows the main vessel partially filled with MICROFIL^®^. **(B)** The 3D volume of the sample B was reported. The inset shows a very intricate collagen matrix (rendered in yellow) coexists with the vessels (light green). The newly formed bone is rendered in light blue. **(C)** The 3D volume of sample C is reported. The segmentation renders the vessels in red and the scaffold in blue. The soft tissues were computationally removed from the 3D rendering to highlight the vessels distribution inside the scaffold. The inset shows in red the numerous vessels crossing the soft tissue (segmented in green). **(D)** The sample D was BMSC seeded but not stained. The vessels are rendered in red, the soft tissue in yellow and green. The inset shows one of the ramified vessels in red.

In order to investigate by XRPCμT the 3D micro-vessels distribution inside the scaffold, a high spatial resolution is required. For this reason, we used the in-line propagation setup of the TOMCAT beamline at SLS, sketched in Figure 1, able to achieve a spatial resolution of 0.64 μm. In the table shown in Figure 1B, we reported the characteristics of the different investigated sample groups. Figure 2 shows details of the vessels distribution inside representative samples from the four different groups implanted for 4 weeks in the mice. The first two samples (A and B) were both stained with MICROFIL^®^, a compound that fills and enhances the opacity of the micro-vascular network. In sample A, no BMSC were seeded on the scaffold, whereas the scaffold of sample B was seeded with BMSC. Sample C was also seeded with BMSC, but, after its recovery from the animal, it was stained with PTA. PTA enhances the soft tissue (ST) signal and thus it was expected to improve the visualization of both the vessels entering the scaffold pores and the collagenous matrix (Campi et al., 2013). Figure 2D displays the tomographic image of the sample D, which was seeded with BMSC (like B and C samples), but neither it was prepared with MICROFIL^®^, nor it underwent staining treatment.

In Figure 2A, the vessels in the sample A are rendered in red, the scaffold in blue, and the ST in yellow and green. The inset of Figure 2A shows a close-up of a vessel filled with MICROFIL^®^ where bone is not formed. Vessels penetrate the scaffold pores, but at first sight a poor branching is already evident. Figure 2B shows the 3D volume of the sample B. The inset of the figure highlights a very intricate matrix (rendered in green) which coexists with the vessels (red) and the newly formed bone (segmented in light blue). Figure 2C shows the 3D volume of sample C. The segmentation renders the vessels in red and the scaffold in blue. Since the PTA enhances the contrast of both vessels and STs, we highlighted the vessels distribution inside the scaffold, by computationally removing the STs from the 3D rendering. The inset shows, in a portion of the volume, the numerous vessels, segmented in red, crossing the ST, which is segmented in green and yellow. Finally, we report in Figure 2D images of sample D. Although the lack of staining makes the visualization of vessels more cumbersome, Figure 2D clearly demonstrates that the imaging of the vascular network without invasive treatments is doable. The vessels are rendered in red, the scaffold in blue, and the ST in yellow and green. The inset shows one of the ramified vessels. We report the 3D rendering of sample B in Video S1 in Supplementary Material.

### Quantitative analysis of tomography images

**Figure 3:**
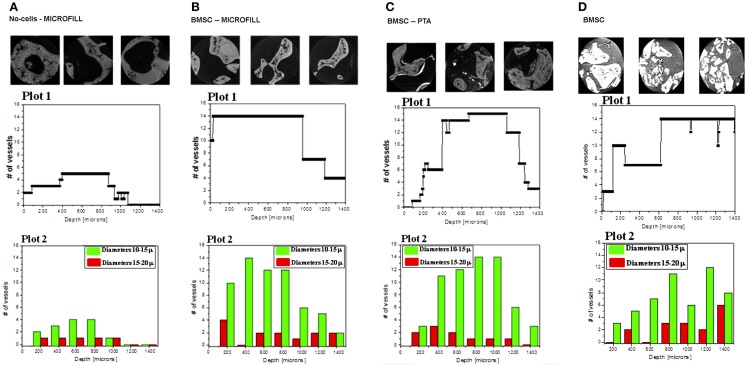
**Quantitative analysis of the A, B, C, and D samples**. Plots 1 show the number of vessels crossing the samples at the different depth inside the volume. For each sample, the three pictures above the plot 1, are the tomography images of the upper, central and top parts of the sample. Plots 2 report the spatial distribution of large vessels in dark (red on line) and small vessels in gray (green on line).

While Figure 2 provides an imaging survey, Figure 3 contains the results of the quantitative analyses. In this figure, we considered the samples A, B, C, and D. We explored a central cube volume of 1.4 mm side, for all the samples. The precision of the selected volume was assured by the precision of the experimental setup, which allowed one to always illuminate equal portions of the samples. Exploiting the 3D character of the tomographic approach, we systematically analyzed the computed sections (each 640 nm thick) of the samples, perpendicular to any chosen direction, studying the number, section, and distribution of the vessels crossing each section. To get insights on the structure of the vascular trees in the recovered implants, we first plotted the number of vessels of the vascular network reaching the system at different depths (Figure 3, plot 1) and then we investigated the depth distribution of vessels with different size (Figure 3, plot 2).

In Figure 3A (sample A), we choose the direction of the large vessel entering the scaffold (appearing from zero in plot 1) and we considered the sections perpendicular to this direction. We could not follow the same approach for the B and C samples since no clear directionality was found in the networks, which seemed more isotropically distributed with high connectivity between the pores in the various directions. The plot 1 reports the total number of vessels (i.e., irrespective from their size) upon varying the depth along the chosen direction. A major difference is visible between the sample A and the other three. In the absence of mesenchymal progenitor cells (sample A), the vascular tree has a poor development and is composed of a single large vessel, which only ramifies from 400 to 800 μm in a few small vessels, before they all disappear in the sample at about 1000 μm. On the contrary, the B, C, and D samples display a wide tree of vessels with several branches covering the whole depth of about 1400 μm in Figure 3B and 1300 μm in Figures 3C,D. The broad distributions of the B, C, and D samples have similar extensions and comparable average values of vessel number.

**Figure 4:**
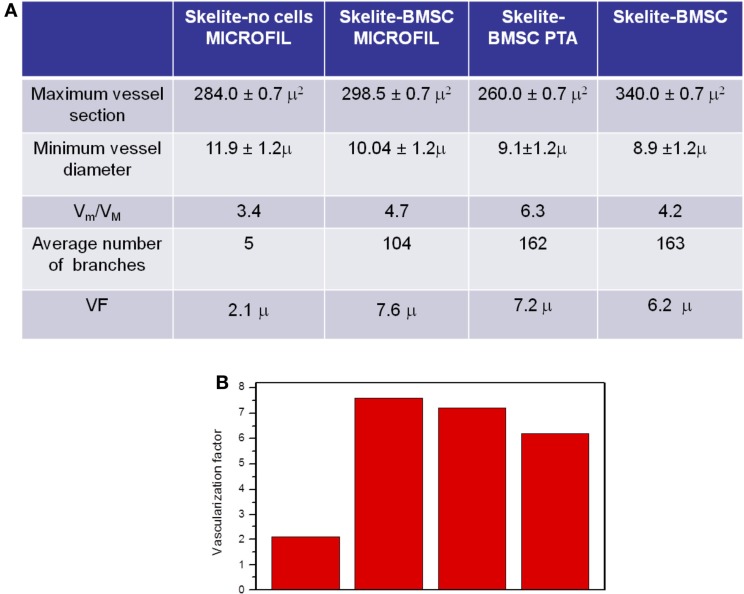
**(A)** Table of quantitative information obtained for the A, B, C, and D samples. V_m_ and V_M_ are the total numbers of small and large vessels, respectively (see text). **(B)** Vascularization factor (see text) for the four samples.

To get higher level of detail on how vessels of different size are spatially distributed, we divided the vessels into two categories: small vessels with a diameter between 10 and 15 μm and large vessels with a diameter between 15 and 20 μm. Plot 2 reports the number V_m_ of small vessels (represented in gray) and the number V_M_ of large vessels (represented in black) at different depths inside the samples. Clearly, in the whole space occupied by the vascular trees of B, C, and D samples, small vessels were by far more numerous and a significant branching occurred. This analysis emphasizes even more strikingly the difference with respect to the A sample, where only one large vessel was present, which intersected the sample, crossing it for about 1 mm, and abruptly disappearing. Few small vessels occupied the first part of the sample, from 400 to 800 μm depth, and then disappeared outside this interval. The table shown in Figure 4A extends and summarizes the quantitative comparison of vessels in the four samples. The individual structure of the vessels was similar in the three samples because the maximal sections and the minimal diameters were comparable. Again the major difference laid in the average number of branches forming the vascular trees. The A sample was poorly ramified, while B, C, and D samples displayed thriving trees with many branches. Finally the overall flow rate of the vascular networks for the four samples, represented by the vascularization factor (VF) reported in the last line of the Table in Figure 4A and visualized in Figure 4B, showed a major difference between sample A and the other three samples. Assuming that for each vessel the blood flow is proportional to its section, this quantity is calculated as the integral of small vessels weighted by their average section (~123 μm^2^) plus the integral of large vessels weighted by their average section (~240 μm^2^) (the sum is then normalized by the total depth of 1400 μm).

### Diffraction analysis

**Figure 5:**
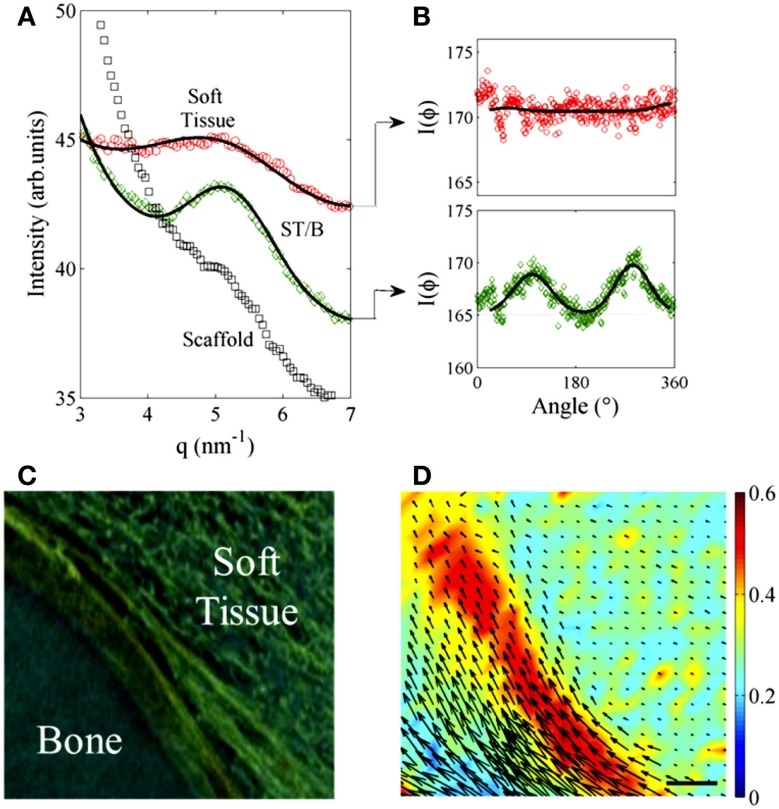
**(A)** Typical diffraction radial profile, I(q), measured for ST (red circles), ST/B interface (green circles), and in the scaffold (black squares). The continuous lines represent the best fitting curves using a Gaussian line shape added to a power law behavior as background. **(B)** Orientation degree of the collagen molecules, given by the area under the peaks of the azimuthal profiles I(Φ), measured in ST (red circles) and at the ST/B interface (green circles). The continuous lines are the Gaussian curve fits above a constant background. **(C)** XRPCμT detail of the ST/B interface. **(D)** Spatial distribution of collagen molecular amount and alignment given by the color intensity and by the arrows vectors, respectively. Size-bar = 15 μm.

Despite the high resolution of the tomographic images, some ambiguity may still be present, like in the insets of Figure 2C where the “hairy” region near the bone might be due to capillaries or to collagenous bundles. To discriminate in the intricate network of “hairy” structures (in green Figure 2) between a capillary network and collagenous oriented fibers, XRμD scanning was already used to explore the time evolution of collagen matrix and structure during the bone mineralization process (Cedola et al., 2006). Here, we used the same technique to confirm that the intricate network was due to collagen fibers matrix. Collagen diffraction gives a broad peak around q = 5.6 nm^−1^, due to the molecular lateral packing. In Figure 5A, we show the typical diffraction radial profiles, I(q), measured in the ST, at the ST/Bone (B) interface and in the scaffold. We could clearly observe how the collagen peak becomes more pronounced as the mineralization arises, i.e., at the ST/B interface. The orientation degree of the collagen molecules is given by the area under the peaks of the azimuthal profiles I(Φ). Typical azimuthal profiles measured in ST and at the ST/B interface, are reported in Figure 5B. Figure 5C shows a typical XRPCμCT image of the ST/B interface, while the spatial distribution of collagen amount and alignment at this interface is reported in Figure 5D. Specifically, the collagen amount, corresponding to the collagen peak area on each measured point, is given by the map intensity; at the same time, the collagen molecules orientation angle and orientation degree are represented by the directions and amplitudes of the black arrows, as in a vector plot. These scanning XRμD results identify the packing and alignment of the collagen molecules close to the newly formed bone far from the scaffold, well distinguished from the (micro)vessels network in the XRPCμT imaging.

## Discussion

The aim of this study was to assess the effectiveness of our XRPCμT approach, combined with XRμD, to study the angiogenesis in engineered bone constructs. The understanding of the angiogenesis in bone-engineered scaffolds still remains an important goal for the regenerative medicine and to allow for the clinical application of this approach. The identification of the most appropriate scaffold for bone tissue regeneration is directly related to the ability of the scaffold to recruit osteoprogenitor cells and endothelial progenitors. On the other hand, new technologies should provide the tools for monitoring angiogenesis in the engineered scaffolds. While traditional histology provides unique insight into tissue morphology, it fails to reveal the spatial organization of the microscopic structures, unless a tedious and time-, resource-, and energy-consuming sectioning s of the whole sample – followed by histological analysis – is performed. In addition, histology and image processing inherently result in small, local tissue artifacts that can introduce errors in the determination of the microscopic tissue structure (Martin et al., 2002). Recently, some authors have demonstrated the possibility to perform an *in vivo* quantification of the vascular network in mice using contrast-enhanced micro-CT (Nebuloni et al., 2014). Although a new phase-contrast medium – the micro-bubble – was recently developed and applied to angiography applications (Tang et al., 2011), different papers have shown that XRPCT is able to perform a 3D visualization of the smallest capillaries (Momose et al., 2000; Fratini et al., 2015) in mice, without the use of any contrast agent.

Within this context, we investigated by synchrotron XRPCμT and XRμD samples that underwent different staining procedures. The first two sets were perfused by the radiopaque medium MICROFIL^®^ (Zagorchev et al., 2010), but only one of these two sets was also seeded with BMSC. The third set was stained with PTA immediately after its explantation from the animal, while the fourth set of samples was left unstained in order to obtain evidence that the imaging of the vascular network was also possible without any previous staining treatment. We used XRPCT because the low-density soft matter making up the collagen fibrils (Campi et al., 2012) and the vessels is not visible via conventional micro-CT based on absorption (Gao et al., 1998; Momose et al., 2000). The high quality achieved for the 3D images enabled the extraction of quantitative pieces of information (number, section, and distribution of the vessels crossing each section) for all the different sample preparations.

The main goal of our work was the technical achievement of 3D imaging of a vascular network. This clearly demonstrated and quantitatively analyzed in the samples where this vascularization was present. Moreover, our data also suggest that seeding the scaffolds with BMSC enforces the vascularization. The major difference lies in the average number of branches forming the vascular trees. The sample not seeded with BMSC was poorly ramified, while the other three samples displayed thriving trees with many branches. It is important to emphasize that the histological studies performed on samples unseeded with cells, show a poor vascularization (Figure S1 in Supplementary Material).

Even though XRPCT was able to visualize the 3D vascularization network inside the scaffold without any sample sectioning and preparation, in order to achieve a higher image quality with sub-micrometer spatial resolution, the use of a coherent, highly brilliant X-ray Synchrotron source was mandatory. This could certainly limit a possible future use of this technique in the clinical routine, but remains a highly valuable experimental approach in pre-clinical researches such as those involving investigation of different scaffold vascularization.

Once the capability of this technique has been established, the way is paved for a wealth of further investigations and more firm biological conclusions can be drawn with more extensive analyses and statistics. In this regard, an obvious future work will be the comparative analysis of different types of scaffolds with different implantation times. We therefore propose our approach as a tool for angiogenesis studies in TE and for any other pre-clinical investigations where the quantitative analysis of the vascular network is required.

## Conflict of Interest Statement

The authors declare that the research was conducted in the absence of any commercial or financial relationships that could be construed as a potential conflict of interest.

## Supplementary Material

The Supplementary Material for this article can be found online at http://journal.frontiersin.org/article/10.3389/fbioe.2015.00133

Figure S1 **Hystological images of engineered Skelite scaffolds stained by MICROFIL^®^**. **(A**,**C**,**E)** scaffold not seeded with cells; **(B**,**D**,**F)** scaffolds seeded with cells. **(A**,**B)** scalebar = 500 μm; **(C**,**D)** scalebar = 200 μm; **(E**,**F)** scalebar = 100 μm. Arrows indicate vessels marked by MICROFIL. Acquisition by Zeiss Axiovert 200M (**sk** = Skelite; **ft** = fibrous tissue; **b** = bone tissue; **m** = MICROFIL^®^).

Video S1 **(a) 3D reconstructions of a volume of about 400 slices of the sample B**. We segmented in red the MICROFIL^®^ and the scaffold, while in green-yellow the soft tissue.
