# Supplementary figures and images for: High-Resolution X-Ray Techniques as New Tool to Investigate the 3D Vascularization of Engineered-Bone Tissue
Source: Front Bioeng Biotechnol. 2015 Sep 7;3:133. doi: 10.3389/fbioe.2015.00133 (PMC4561513; doi:10.3389/fbioe.2015.00133)

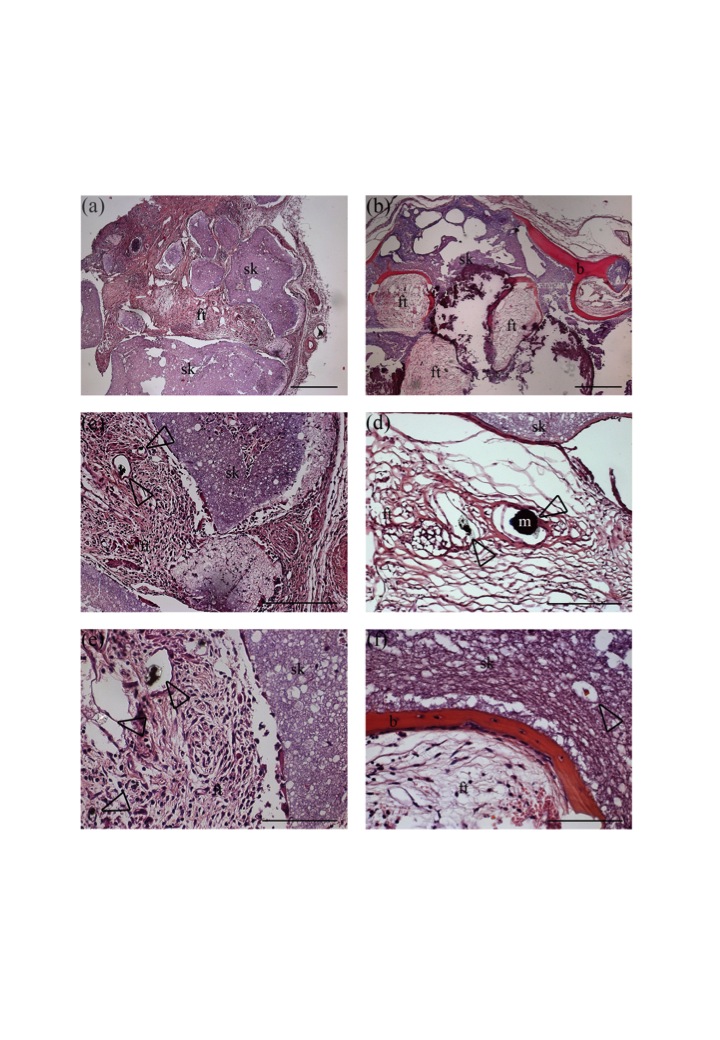

Supplement: Figure S1 — Hystological images of engineered Skelite scaffolds stained by MICROFIL®. (A,C,E) scaffold not seeded with cells; (B,D,F) scaffolds seeded with cells. (A,B) scalebar = 500 μm; (C,D) scalebar = 200 μm; (E,F) scalebar = 100 μm. Arrows indicate vessels marked by MICROFIL. Acquisition by Zeiss Axiovert 200M (sk = Skelite; ft = fibrous tissue; b = bone tissue; m = MICROFIL®). [file Image_1.JPEG]
